# Neglected vaginal foreign body leading to vaginolith, vesicovaginal fistula and vesical calculus formation in an adolescent girl

**DOI:** 10.1259/bjrcr.20150474

**Published:** 2016-05-08

**Authors:** Mukesh Surya, Chanderdeep Sharma, Dinesh Sood, Anjali Soni, Rajkumar Sharma, Khanak Nandolia

**Affiliations:** ^1^ Department of Radiodiagnosis, DRPGMC Kangra at Tanda, Kangra, India; ^2^ Department of Obstetrics & Gynaecology, DRPGMC Kangra at Tanda, Kangra, India; ^3^ Department of Surgery, DRPGMC Kangra at Tanda, Kangra, India

## Abstract

Vaginal foreign body insertion is not an uncommon clinical entity; however, long-standing neglected vaginal foreign body causing vaginolith, vesicovaginal fistula and vesical calculus formation is unusual. We present a case of a neglected vaginal foreign body (plastic cap of a nail colour) leading to vesicovaginal fistula, vaginolith and vesical calculus formation in a 12-year-old female child presenting with continuous dribbling of urine per vagina. Diagnosis was confirmed on ultrasonography, non-contrast CT scan and MRI of the pelvis. The MRI demonstrated the exact size and site of the urinary bladder wall defect, besides the foreign body and the vesical calculus. The foreign body along with vaginolith and the vesical calculus were removed *via* suprapubic approach under general anaesthesia; the fistula was repaired by suturing the urinary bladder and vagina wall defect.

## Case

A 12-year-old female child presented to the gynaecology outpatient department with complaints of continuous dribbling of urine for the past 6 months. She had not attained menarche and had a history of fall and pelvic injury 3 years back. Her general physical examination was unremarkable. On local examination of the external genitalia, there was marked excoriation of the perineal and vulval skin. Foul smelling urine was observed coming out of the vaginal opening. Her routine laboratory investigations such as haemoglobin, total leukocyte count, differential leukocyte count, erythrocyte sedimentation rate, blood urea and creatinine were within normal limits. Based on the history and clinical examination, a provisional clinical diagnosis of post-traumatic vesicovaginal fistula (VVF) was made and the patient was referred to the radiology department for ultrasonography (USG) of the kidneys, ureters and bladder.

**Figure 1.**
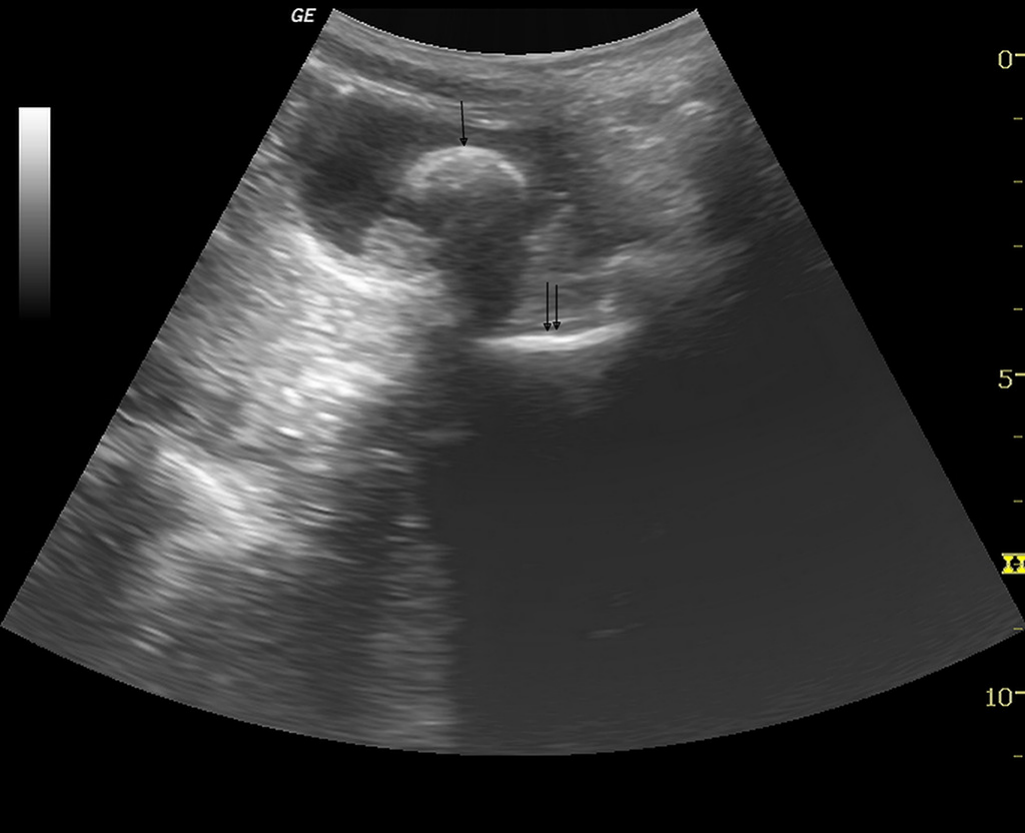
Sagittal sonogram of the pelvis showing a large calculus (arrow) in the lumen of the urinary bladder. Another echogenic shadow (double arrow) is seen posterior to the urinary bladder, suggestive of a vaginal foreign body.

**Figure 2.**
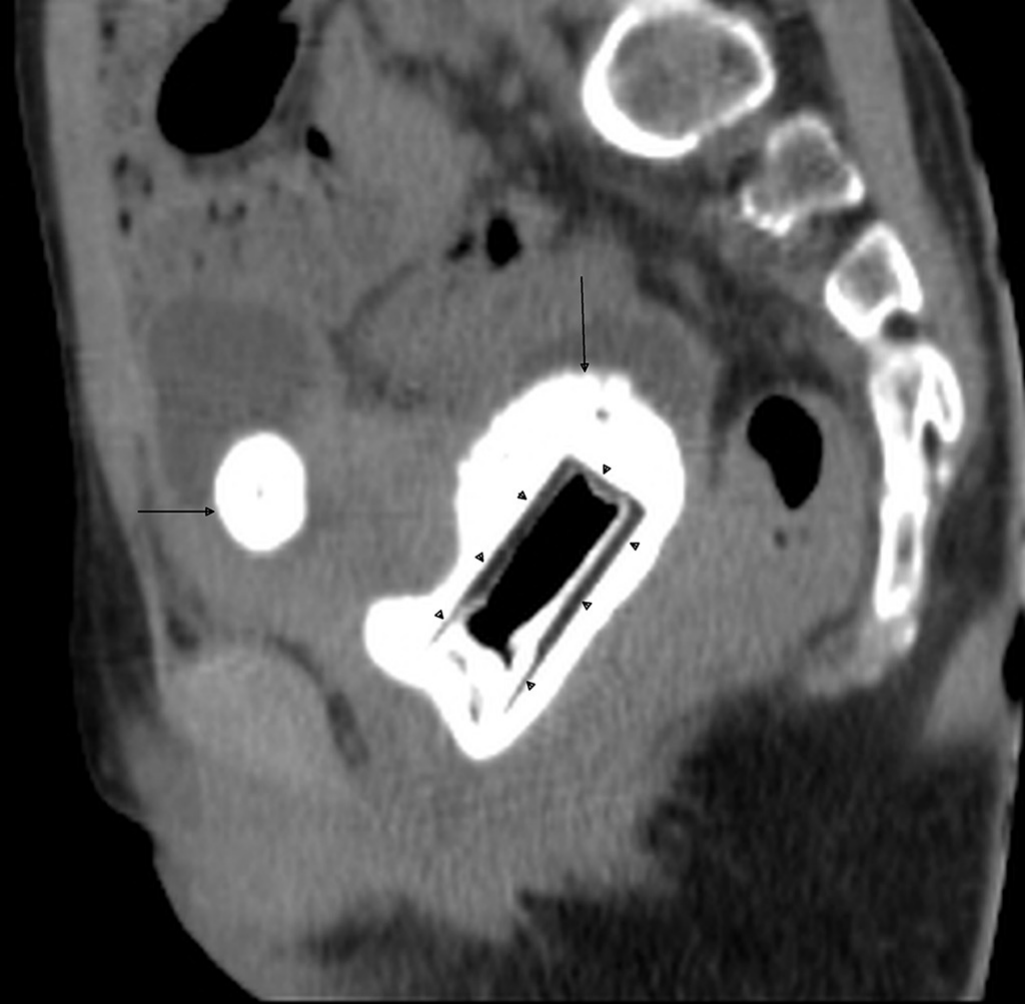
Sagittal reformatted non-contrast CT scan of the pelvis showing a big vaginolith (vertical arrow) surrounding the foreign body (arrowheads). Vesical calculus (horizontal arrow) is also seen in the lumen of the urinary bladder.

**Figure 3.**
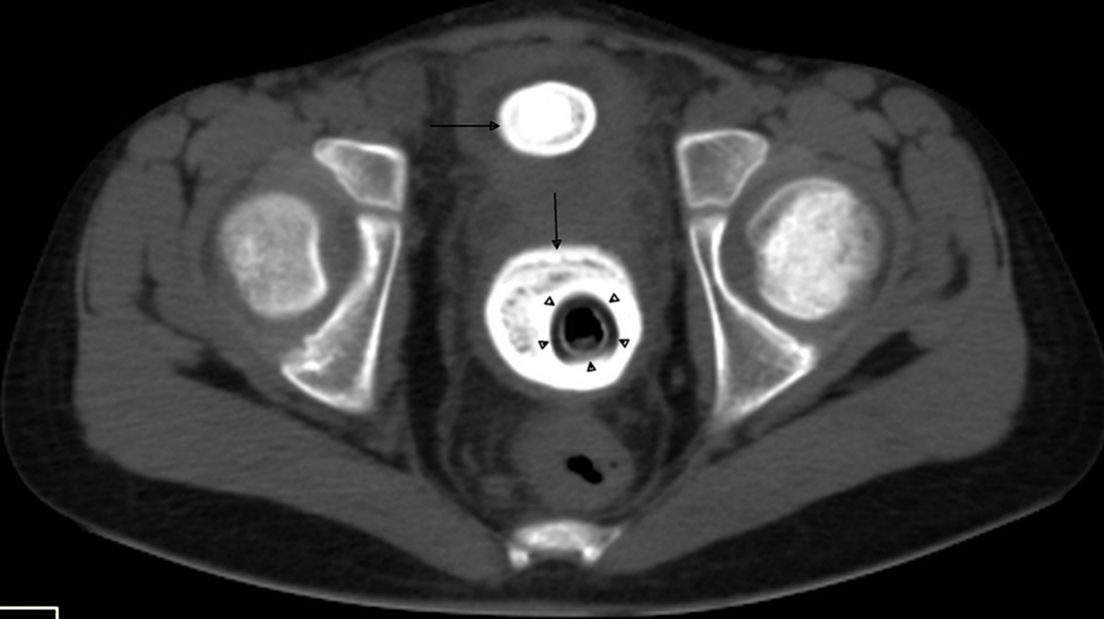
Axial non-contrast CT scan of the pelvis showing a vesical calculus (horizontal arrow) and a vaginolith (vertical arrow) surrounding the vaginal foreign body (arrowheads).

USG was performed on a GE Logiq P5 machine (General Electronics Co., Milwaukee, WI) and revealed mild bilateral hydronephrosis. A curved echogenic focus with posterior acoustic shadow was seen within the lumen of the partially distended urinary bladder measuring approximately 25 mm in size, suggestive of a vesical calculus (single arrow in Figure 1). Another echogenic shadow was seen between the urinary bladder and the rectum, raising suspicion of a vaginal foreign body (double arrow in Figure 1). When enquired, the patient and her parents denied the insertion of any foreign body into the vagina. The child was taken for an urgent non-contrast CT scan of the pelvis for confirmation of the USG findings. The CT was performed on a Philips Brilliance 16 machine (Phillips Healthcare, Amsterdam, Netherlands), which revealed a calculus of size 28 × 25 × 18 mm in the lumen of the urinary bladder (horizontal arrows in Figures 2 and 3). A big rectangular calcified mass was seen in the vagina measuring 53 × 29 × 33 mm in size (vertical arrows in Figures 2 and 3). A well-defined rectangular hypodense rim was noted inside the calcified mass measuring 36 × 18 mm in size (arrowheads in Figures 2 and 3), containing an air dense area in the centre. The patient was again asked about the vaginal foreign body and she admitted to inserting the plastic cap of a nail colour into her vagina about a year back.

**Figure 4.**
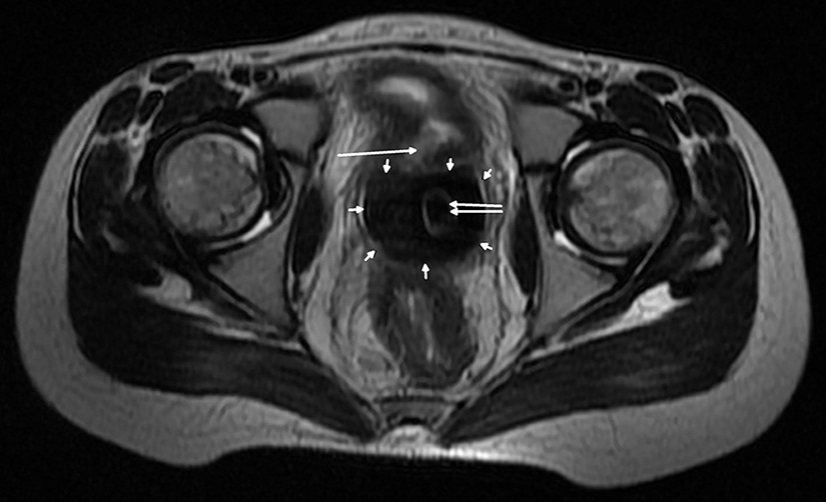
Axial *T*_2_ weighted fast spin-echo MRI showing a defect (large arrow) in the posterior wall of the urinary bladder. A hypointense vaginolith (multiple small arrows) is seen around intermediate signal intensity vaginal foreign body (double arrow).

**Figure 5.**
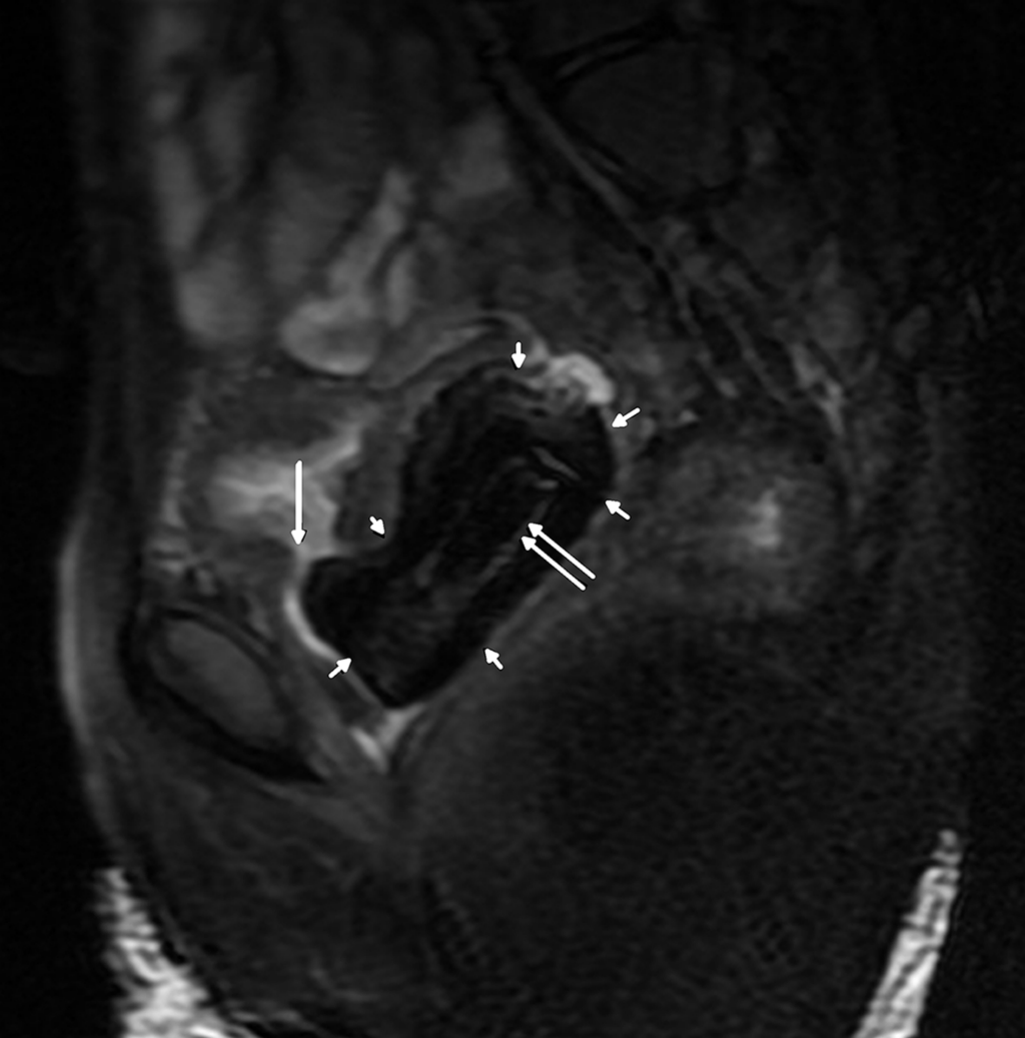
Fat-saturated *T*_2_ weighted fast spin-echo sagittal MRI showing a large defect in the posterior wall of the urinary bladder (large arrow). An intermediate signal intensity foreign body (double arrow) surrounded by hypointense vaginolith (multiple small arrows) is also seen.

An MRI of the pelvic organs was performed the next day on a 1.5 T GE Signa Excite (General Electronics Co.) for detailed evaluation. The following sequences were employed: sagittal *T*_2_ weighted fast spin echo (FSE) [repetition time (TR) 4243 ms, echo time (TE) 63 ms]; sagittal *T*_2_ weighted fat-saturated (FS) FSE (TR 4600 ms, TE 63.3 ms); sagittal *T*_1_ weighted FSE (TR 500 ms, TE 8 ms); coronal *T*_2_ weighted FS-FSE (TR 4600 ms, TE 63.3 ms); coronal *T*_2_ weighted FSE (TR 4200 ms, TE 63.3 ms); coronal *T*_1_ weighted FSE (TR 520 ms, TE 8 ms); axial *T*_2_ weighted FSE (TR 5020 ms, TE 106.2 ms); axial short tau inversion-recovery (TR 6260 ms, TE 55.8 ms, inversion time 150 ms); axial *T*_1_ weighted FSE (TR 440 ms, TE 9.1 ms); axial post-intravenous gadolinium *T*_1_ weighted FS-FSE (TR 900 ms, TE 9.1 ms); coronal *T*_1_ weighted FS-FSE (TR 760 ms, TE 7.9 ms); sagittal *T*_1_ weighted FS-FSE (TR 720 ms, TE 8.7 ms). A big vaginolith measuring 52 × 28 × 35 mm in size was seen in the vagina, which was hypointense on all pulse sequences (multiple small arrows in Figures 4 and 5). A well-defined rectangular rim of intermediate signal intensity was seen inside the hypointense mass, suggesting the presence of a foreign body (double arrows in Figures 4 and 5). A defect measuring approximately 6 × 5 mm in size was observed in the posterior wall of the urinary bladder, with free passage of urine into the vagina (single arrows in Figures 4 and 5). A well-defined *T*_1_ and *T*_2_ weighted hypointense calculus was seen in the lumen of the partially distended urinary bladder (23 × 16 × 25 mm).

Based on the radiological findings and history, a diagnosis of vaginal foreign body with secondary vaginolith with vesical calculus with VVF was made. The patient was given antibiotic cover. The vaginal foreign body and the vesical calculus were removed *via* a suprapubic approach under general anaesthesia. The VVF was repaired by suturing the vaginal and bladder wall defect. The patient had an uneventful postoperative stay. The child is healthy and asymptomatic after 3-month follow-up.

## Discussion

Foreign body insertion into the vagina is not uncommon. Extraction of a wide variety of vaginal foreign bodies such as plastic toys and cups, pins, aerosol caps, screws, crayons, coins and sanitary tampons has been reported in the literature.^1^ Children usually push different objects into the vagina out of curiosity while exploring their body. Sexual gratification or contraception is the main objective of foreign body insertion in adult females. In our case, the child inserted the plastic cap of a nail colour just for the sake of curiosity. She could not remove it on her own, but did not tell her parents because of embarrassment and fear of being reprimanded.

Various complications of long-standing vaginal foreign body include sepsis, bleeding per vagina and stenosis. Rarely, it may lead to pressure necrosis, ulceration and perforation of the vaginal wall, ultimately resulting in the formation of a fistula.^2^ Vesicovaginal, rectovaginal, urethrovaginal and ureterovaginal fistulas have been reported in the literature. In our patient, the sharp edges of the foreign body might have led to ulceration and fistula formation between the vagina and the urinary bladder. Deposition of urinary salts on the foreign body caused secondary vaginolith formation. Pressure of the foreign body on the urinary bladder neck led to partial bladder outlet obstruction, causing stasis and, ultimately, calculus formation.

Obstetric injury, gynaecological surgery, pelvic malignancy, radiation therapy and trauma are common causes of VVF formation,^3^ while vaginal foreign body is a rare cause. Very few cases of vaginal foreign body causing VVF have been reported in the literature.^4^ However, to the best of our knowledge, this is the first case of neglected foreign body leading to vaginolith, VVF and vesical calculus formation.

The diagnosis of vaginal foreign body may be difficult, especially when the patient denies any history of foreign body insertion, as in our case. Plain X-ray may reveal the presence of metallic and radiopaque foreign bodies. USG can demonstrate associated complications such as cystitis and back pressure changes in the kidneys. CT and CT cystography are the common radiological investigations being performed at the present time for localization of a foreign body and its complications such as VVF. MRI is rarely used for diagnosing a vaginal foreign body. We could find only one case report where MRI had been used to diagnose a vaginal foreign body in a 7-year-old child.^5^ On MRI, most of the foreign bodies, vesical calculi and vaginolith appear as hypointense structures. Moreover, MRI is best suited for better evaluation of the exact size and site of defects in the wall of the urinary bladder, if any, owing to its better soft tissue contrast resolution. Thus, MRI can furnish additional information in complicated cases, particularly in children. We advocate the following pulse sequences: sagittal *T*_2_ weighted FSE; sagittal *T*_2_ weighted FS-FSE; sagittal *T*_1_ weighted FSE; coronal *T*_2_ weighted FS-FSE; coronal *T*_2_ weighted FSE; coronal *T*_1_ weighted FSE; axial *T*_2_ weighted FSE; axial short tau inversion-recovery; axial *T*_1_ weighted FSE; axial post-intravenous gadolinium *T*_1_ weighted FS-FSE; coronal *T*_1_ weighted FS-FSE; sagittal *T*_1_ weighted FS-FSE. Delayed post-contrast scans may be helpful for visualization of fistula tract and pooling of contrast in the vagina.

Treatment of vaginal foreign body is removal of the foreign body under local or general anaesthesia. A transvaginal approach is preferred; however, difficult cases may require a transabdominal approach. The VVF is repaired when sepsis is controlled. However, some authors advocate immediate repair of VVF.

## Learning points

1. Neglected vaginal foreign body may lead to serious complications such as VVF formation.
2. Diagnosis of vaginal foreign body may be challenging, especially when the patient is unaware or denies knowledge of the vaginal foreign body.
3. Plain radiography and CT scan are commonly used radiological investigations for confirmation of vaginal foreign body. MRI may be useful for evaluation of associated complications owing to its better soft tissue contrast resolution.
4. Removal of foreign body under local or general anaesthesia is the treatment of choice. Associated complications such as VVF are managed by transvaginal or transvesical repair of the defect, in the same sitting or later.

## Consent

Informed consent was obtained from the patient and her parents.
